# Graphene Based Controllable Broadband Terahertz Metamaterial Absorber with Transmission Band

**DOI:** 10.3390/ma11122409

**Published:** 2018-11-29

**Authors:** Qihui Zhou, Song Zha, Peiguo Liu, Chenxi Liu, Li-an Bian, Jihong Zhang, Hanqing Liu, Liang Ding

**Affiliations:** 1 College of Electronic Science, National University of Defense Technology, Changsha 410073, China; zhouqihui10@126.com (Q.Z.); pg731@126.com (P.L.); liuchenxi09@nudt.edu.cn (C.L.); dk061bianlian@126.com (L.B.); zhangjihong11@nudt.edu.cn (J.Z.); curryhq1995@163.com (H.L.); lio.dingliang@hotmail.com (L.D.); 2 School of Physical and Electronic Science, Changsha University of Science and Technology, Changsha 410114, China

**Keywords:** metamaterial absorber, graphene, broadband absorption, passband

## Abstract

A graphene-based controllable broadband terahertz metamaterial absorber with transmission band is presented in this paper. It consists of a graphene-SiO_2_-frequency selective surface (FSS) sandwich structure. The sinusoidal graphene layer supports continuous plasmonic resonances, forming a broad electric-tuning absorbing band. Bandpass FSS constructs a transmission window outside the absorbing band. The simulation results indicate that the absorption from 0.5 THz to 1 THz can be tuned continuously from 0.4 to 0.9 with angle and polarization independence. A transparent window peaking at 1.65 THz maintains high transmittance over 0.7. The metamaterial absorber has potential applications for detection, stealth, filtering, and electromagnetic compatibility.

## 1. Introduction

Terahertz (THz) absorbers have aroused the interest of more and more researchers due to potential applications for detection, sensing, trapping, communication, and stealth [1,2,3,4,5]. The absorbers, constructed by various structures, such as Salisbury screen and circuit analog absorber, confine the incident wave to the lossy materials to achieve high absorption. However, the operation performances of absorbers are fixed once fabricated. Many traditional controlling methods, such as loading lumped elements, are inapplicable in THz because of the restriction of size [6]. Owing to the unique properties, including excellent mechanical properties, the capacity to support surface plasmon polaritons (SPPs), and the tunability of its carrier mobility and conductivity, graphene is one of the promising materials for designing tunable absorbers in THz region [7,8,9,10]. Since the absorption of single undoped graphene is just close to 2.3%, a great many of THz metamaterial absorbers (TMAs) based on patterned graphene have been put forward to improve the absorption intensity, such as nanodisks, microribbons, fishnet and so on [11,12,13].

To sustain the SPPs, the operating bandwidth is generally narrow, which limits the application scope of TMAs. Therefore, the methods to widen the bandwidth have received considerable attention. One means of overcoming this limitation is by integrating several resonators with similar shapes and different sizes into a unit cell so that adjacent resonance peaks are overlapped, thus, forming a broad absorbing band [14,15]. Another way is to bias stacked patterned graphene layers separated by dielectric substrates at different voltages to expand the bandwidth [16,17]. Recently, the idea of utilizing the single-layered graphene with gradient width structure, such as ellipse or sinusoid, to excite continuous plasmon resonances realizes broad absorbing band without complex design [18,19,20]. However, it is difficult to bias the multi-resonator/multi-layered structures with different biasing voltages and avoid the coupling between resonators or layers. In addition, most of the TMAs with gradient width are polarization dependent. Moreover, to greatly improve the absorption, TMAs mentioned above usually adopt metal plate to reflect all of the incident waves so that the requirements of some practical applications cannot be satisfied. For example, electromagnetic compatibility and filtering both need broadband absorption along with a transmission band to ensure the propagation of normal signals.

Here we study the electromagnetic response of a composite structure consisting of a single sinusoidal-patterned graphene layer deposited on a SiO_2_ dielectric layer backed with a bandpass frequency selective surface (FSS), which achieves a controllable broad absorbing band with a transmission window. Compared with our previous works in Reference [20], the TMA not only solves the problem of polarization independence and electrical control of the chemical potential but also introduces an extra transmission band. The structural improvement on sinusoidal graphene directly excites continuous plasmon resonances to achieve broadband absorption in the terahertz region and ensures the incident angle and polarization independence. Tuning the chemical potential *E_F_* via a voltage bias, the absorption can be regulated continuously. Moreover, adopting a bandpass FSS to substitute the gold ground allows the signals in the passband to transmit with low attenuation and reflects transmitted waves in the absorbing band without affecting the performance of TMA.

The device can be used as an amplitude modulator. When the incident wave operating at the absorbing band impinges obliquely on TMA, the amplitude of the reflected wave can be regulated continuously via tuning the voltage between graphene and FSS to control the absorptivity. The angle independence makes the separation of incident and reflected waves more convenient. In addition, in the telecommunication system, the TMA can function as a powerful spatial filter. It not only absorbs the low-frequency signal with high penetration but also ensures the normal operating signal passes with little loss, which contributes to the anti-interference, electromagnetic compatibility, and signal separation. Moreover, the power of incident wave can be collected by probe and transformed into voltage between the graphene and FSS. Meanwhile, the absorption changes accordingly, and the power of the incident wave is sensed by the TMA. It should be noted that the design scheme can be applied to other frequency regions due to the scalability of metamaterial and the idea of replacing metal ground with FSS is applicable for other kinds of TMA to expand the practical scope.

## 2. Materials and Methods

**Figure 1:**
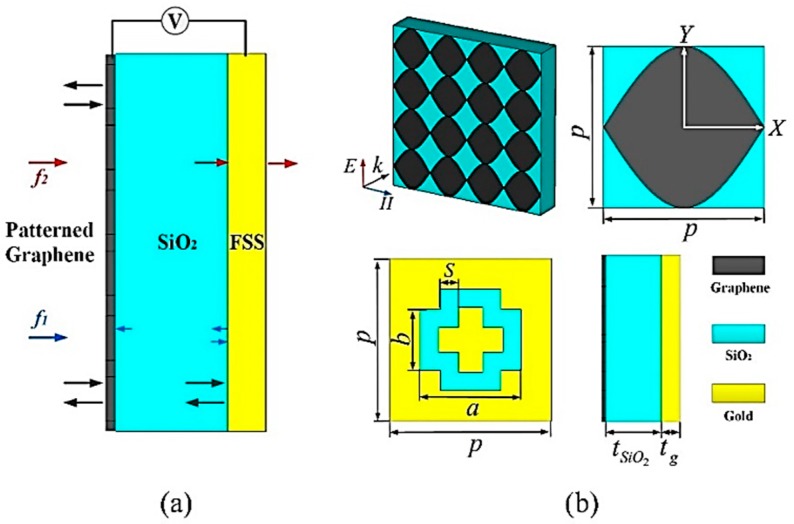
Structure of the controllable terahertz metamaterial absorbers (TMA). (**a**) Profile sketch, (**b**) unit cell. The dimension values are set as *p* = 80, *a* = 50, *b* = 30, *s* = 10, *t_SiO2_* = 55, *t_g_* = 0.5, unit: μm.

As indicated in Figure 1a, the proposed TMA is composed of three parts: a patterned graphene on the top, a SiO_2_ spacer in the middle, and a gold bandpass FSS at the bottom. The pattern of graphene consists of two intersecting sinusoidal curves, the functions of which are expressed as follows:

(1) $$y_{1}=\frac{p}{2}\cos\frac{\pi }{p}x,\;y_{2}=-\frac{p}{2}\cos\frac{\pi }{p}x$$

where *p* represents the period of the unit cell. The gradually-changed widths support continuous plasmonic resonances excited by the incident wave, thus, forming a broad absorbing band *f*_1_. The plasmonic resonances of graphene have polarization independent property and the same period in both *x* and *y* directions ensures the polarization independence. The SiO_2_ (*ε_SiO2_* = 4, [21]) with thickness *t_SiO2_*, which is a favorable substrate to support the graphene film, acts as the spacer. The bandpass FSS plays the role of ground to sustain high absorption at *f*_1_ while has little attenuation at its resonant frequency *f*_2_, thus, forming an extra transmission window. In this case, we take a cross-slot array to generate LC (capacitor and inductor) parallel resonance so as to construct a passband [22]. The period, outer length, outer width, and slot width of the FSS are represented with *p*, *a*, *b*, and *s*, respectively. Gold with thickness *t_g_* is modeled as a lossy metal material with a conductivity of 4.56 × 10^7^ S/m [23]. Controlling the voltage between the graphene and FSS, the absorption at *f*_1_ is regulated since the chemical potential *E_F_* tuned by the voltage changes the conductivity of the graphene layer.

The control of absorption should be contributed to the tunability of graphene. Graphene is modelled as infinitely thin layer described by surface impedance [24,25]. The surface conductivity consists of inter-band and intra-band transitions, which can be calculated through Kubo formula [26,27]:

(2) $$\sigma_{g}=\sigma_{g}^{intra}+\sigma_{g}^{inter}$$

(3) $$\sigma_{g}^{intra}=\frac{2k_{B}Te^{2}}{\pi \hbar^{2}}\ln\left[2\cosh\left(\frac{E_{F}}{2k_{B}T}\right)\right]\frac{i}{\omega +i\tau^{-1}}$$

(4) $$\sigma_{g}^{inter}=\frac{e^{2}}{4\hbar }\left[H\left(\omega /2\right)+i\frac{4\omega }{\pi }\int_{0}^{\infty }\frac{H\left(\Omega \right)-H\left(\omega /2\right)}{\omega^{2}-4\Omega^{2}}d\Omega \right]$$

where *k_B_* is the Boltzmann’s constant, *e* represents the charge of an electron and *ℏ* = *h/2π* denotes the reduced Planck’s constant, respectively. *E_F_* is the chemical potential (Fermi energy), *τ* is the relaxation time set as 0.3 ps, *T* is room temperature set as 300 K and *H*(Ω) is defined as:

(5) $$H\left(\Omega \right)=\frac{\sinh\left(\hbar \Omega /k_{B}T\right)}{\cosh\left(E_{F}/k_{B}T\right)+\cosh\left(\hbar \Omega /k_{B}T\right)}$$

considering the Pauli exclusion principle, the inter-band contribution to graphene conductivity is negligible in the low THz region. *E_F_* is regulatable via controlling the bias voltage between patterned graphene and FSS, thus, changing the conductivity of graphene, resulting in the variation of resonant depth of TMA. The reflectance and transmittance are obtained by *R* = |S_11_|^2^, *T* = |S_21_|^2^ (S_11_: reflection coefficient, S_21_: transmission coefficient). And the absorption is calculated by *A* = 1 – *R* − *T*. All the numerical simulations are carried out by CST Microwave Studio in this paper.

## 3. Results and Discussion

**Figure 2:**
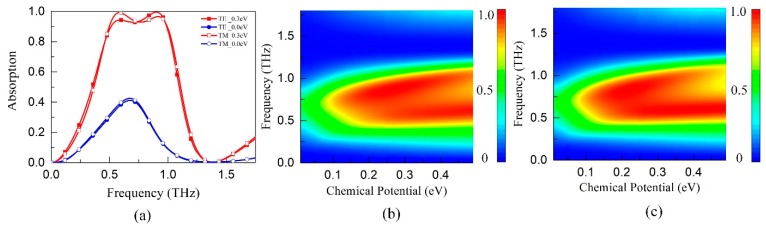
Absorption spectrums of the controllable TMA backed with gold film (**a**) *E_F_* = 0.3 eV, TE (transverse electrical) and TM (transverse magnetic); (**b**) as a function of frequency and *E_F_*, TE; (**c**) as a function of frequency and *E_F_*, TM.

First, we simulate the broadband TMA backed with gold film instead of the FSS to better understand the principle and simply the analysis. Figure 2a compares the absorption curves in TE (transverse electrical) and TM (transverse magnetic) polarizations under normal incidence. When *E_F_* = 0.3 eV, the effective absorption band (absorption > 0.9) ranges from 0.5 THz to 1.0 THz, with a normalized bandwidth of 66.7%. The maximum absorption drops to less than 0.4 as the *E_F_* decreases to 0 eV so that a high switching intensity (>50%) can be achieved by electrical control of the graphene. The operating band of TM polarization almost coincided with that of TE polarization since the width variation ranges, which depend on the resonance length and operating band, are the same in both x and y directions. But there are still some small, inconsistent fluctuations in the effective absorption band, which is mainly caused by the different curvature variation of the unit cell in x and y directions. Moreover, the absorption spectrums as a function of the frequency and *E_F_* for both TE and TM polarizations have been calculated and displayed in Figure 2b,c, respectively. As *E_F_* increases from 0 eV to 0.5 eV, the absorption is improved gradually and then shows saturate tendency with operating band unchanged. The simulation results show excellent polarization independence and controllable absorption property.

**Figure 3:**
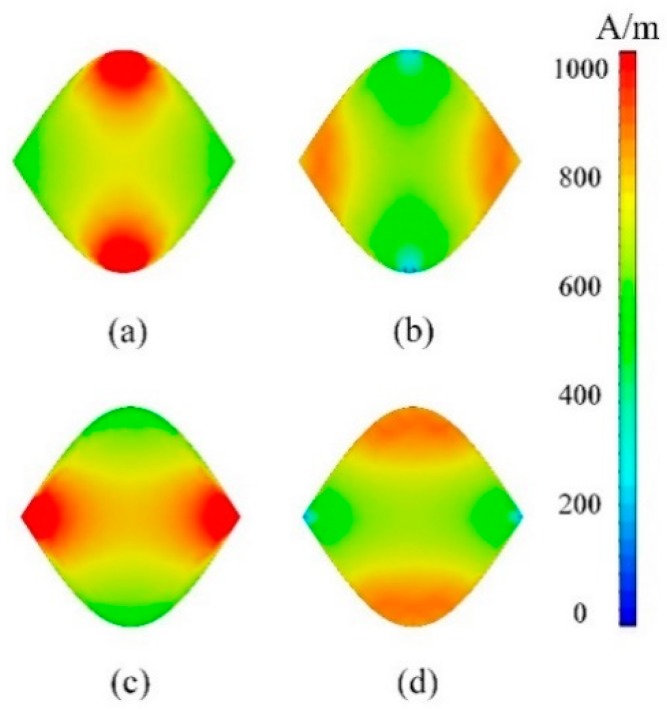
Distributions of surface current intensity of the TMA. (**a**) 0.5 THz, TE; (**b**) 1 THz, TE; (**c**) 0.5 THz, TM; (**d**) 1 THz, TM.

To better understand the principle of broadband absorption, the distributions of surface current intensity are plotted in Figure 3. Figure 3a,b shows the surface current intensity for TE polarization at 0.5 THz and 1 THz. The surface current excited by the incident wave is distributed along the y direction, consistent with the direction of the incident electric vector. In addition, it is concentrated in the center part with maximum resonance length at 0.5 THz while at the end of the unit with minimum resonant length at 1 THz. As for TM polarization, it is indicated in Figure 3c,d that the distribution of surface current is perpendicular to that of TE polarization with the similar regularity due to the change of incident electric field direction. It can be found that the plasmon resonance frequency of graphene increases obviously with the decrease in width, in accordance with the theoretical analysis of Reference [18]. Therefore, the continuous resonant peaks triggered by the gradient width are overlapped with each other, thus, constructing a broad absorbing band.

**Figure 4:**
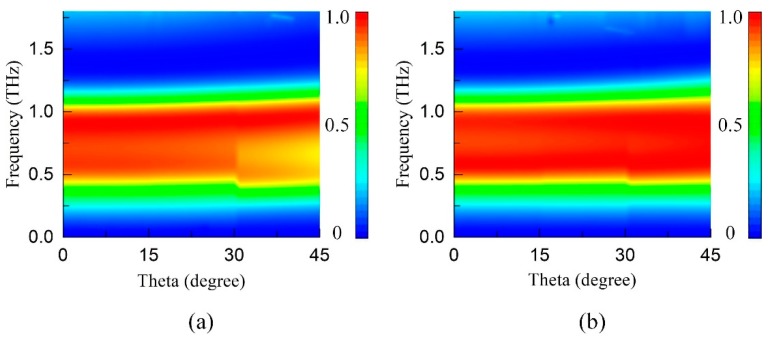
Absorption of the TMA as a function of frequency and incident angle. (**a**) TE, (**b**) TM.

Except the tunability, the angle independence is also one of the important characteristics for TMAs exploiting plasmon resonance of graphene [18]. Figure 4 illustrates the absorption spectrums as a function of frequency and incident wave for both TE and TM polarizations. It can be seen that the incident-angle absorption dependence is relatively weak when the incident angle varies between 0° and 45°. From Figure 2a, we can see that the absorption is relatively small at the low absorbing band for TE polarization and high absorbing band for TM polarization. Both two resonant points are corresponding to the peak position of the sinusoidal curve where the curvature is relatively small and the adjacent elements are close to each other. Therefore, the distribution of power is more dispersed. As the incident angle increases gradually, the field component exciting plasmon resonance decreases so that the absorption will drop as well. However, according to the Reference [19], there will be some parasitic resonances to enhance the absorption in the high region of absorbing band. Therefore, the absorption spectrum decreases in the lower region of absorbing band for TE polarization while it has no obvious decline for TM polarization. Although the absorption decreases for TE polarization in 0.5 THz to 0.8 THz when the incident angle increases from 30° to 45°, the general absorption is still over 0.8. The overall absorption effect is not affected. The angle independence of TMA has great values in practical application of detection and stealth.

**Figure 5:**
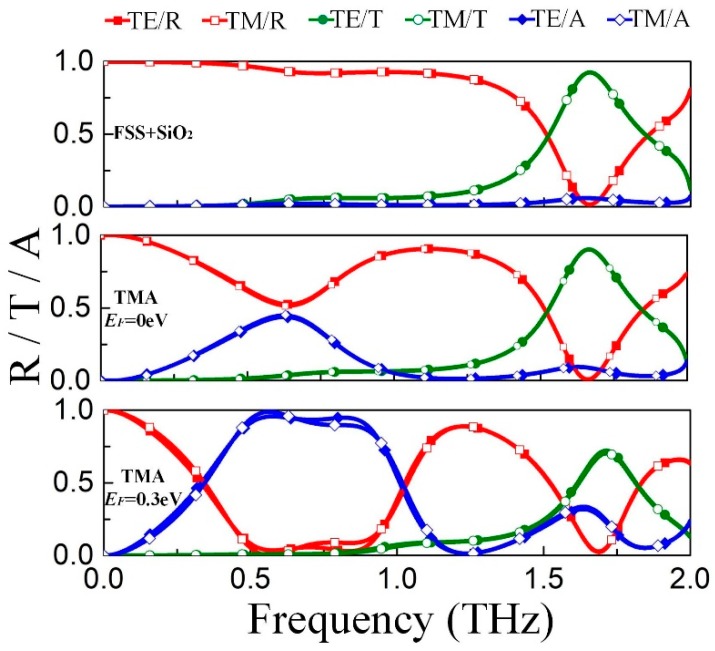
Reflection, transmission and absorption through the frequency selective surface (FSS) and the TMA backed with FSS. **Top**: FSS integrated with substrate; **middle**: TMA, *E_F_* = 0 eV; **bottom**: TMA, *E_F_* = 0.3 eV.

Based on the angle and polarization independent TMA, we adopt the bandpass FSS to replace the gold ground so that an extra transmission band takes shape, thus, expanding fields of application. In the integration design, the substrate in front of the FSS leads to the drop of resonant frequency [22]. Therefore, the FSS is simulated with the SiO_2_ substrate, and the spectrum response is displaced at the top of Figure 5. There is a transparent window with transmittance more than 0.92 at 1.65 THz, and the C4 symmetric of unit cell ensures polarization independence. From 0.5 THz to 1 THz, the reflectance approaches 1, which means that the FSS can effectively act as the reflector of TMA. Substituting the gold film with the bandpass FSS, the spectrum response of proposed TMA is shown in Figure 5. As *E_F_* = 0 eV, a partial absorbing band with 0.4 absorption peaks at around 0.63 THz and the passband locating at 1.65 THz still sustains transmittance over 0.9. As *E_F_* increases to 0.3 eV, there is a broad effective absorption band ranging from 0.5 THz to 1 THz, but the transmission of the passband decreases to 0.7. As illustrated in Figure 2a, the additional transmission loss results from the gradually-increasing absorption of graphene layer above 1.5 THz, which may be eliminated by optimizing the structure dimensions of TMA. To summarize, the spectrum of the TMA backed with FSS is superposed by that of TMA backed with gold film and that of the bandpass FSS. This method expands the application scope of TMA where extra transmission window is required, such as electromagnetic compatibility and filtering.

**Figure 6:**
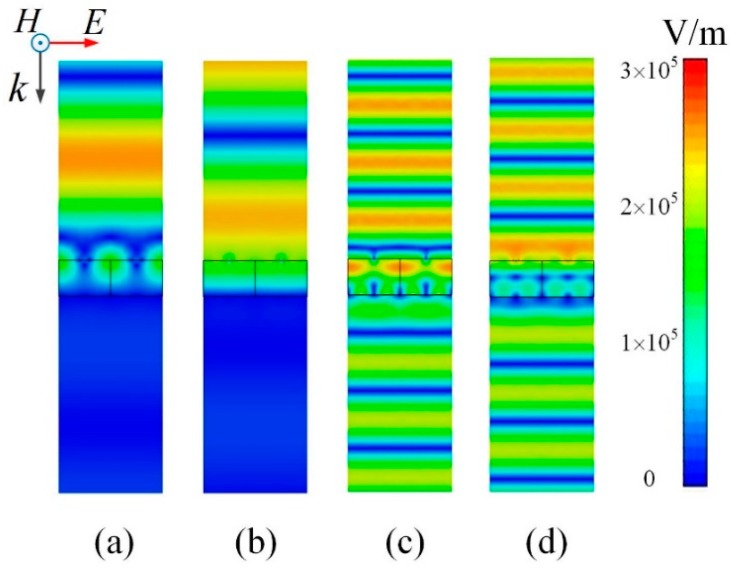
Electric field intensity distributions along the propagation direction when *E_F_* = 0.3 eV. (**a**) 0.6 THz, Phase = 0°; (**b**) 0.6 THz, Phase = 90°; (**c**) 1.65 THz, Phase = 0°; (**d**) 1.65 THz, Phase = 90°.

Further, for TE polarization, the electric field intensity distributions along the propagation direction at 0.6 THz and 1.65 THz are given in Figure 6 when *E_F_* = 0.3 eV. As shown in Figure 6a,b, when the phase of the incident wave increases from 0° to 90°, the maximum intensity of electric field remains unchanged while the position of wave peak moves towards the TMA at 0.6 THz, which means there is a traveling wave in the upper half space. Due to the traveling wave above the graphene layer and transmission towards zero below the FSS, almost all of the incident waves are absorbed by the TMA. As for 1.65 THz, Figure 6c,d shows a traveling wave in both upper and low half space, implying that the incident wave passes through the TMA without obstacles. However, the electric field intensity of transmitted wave shows slight attenuation, which is caused by the loss of the patterned graphene layer at 1.65 THz. In general, the TMA realizes a controllable broad absorbing band and a fixed transmission band with a patterned graphene-SiO_2_-FSS sandwich structure.

## 4. Conclusions

In summary, we demonstrate a tunable broadband metamaterial absorber with a transmission window, which is constituted by a sinusoidal graphene layer and a gold FSS separated by a SiO_2_ substrate. The angle-independent broadband absorption is formed by continuous plasmonic resonances supported by the gradient widths, and the same period in both *x* and *y* directions ensures the polarization independence. The absorptivity can be tuned from 0.4 to 0.9 over 0.5 THz to 1 THz via electrostatically controlling chemical potentials of graphene. Substituting the traditional metal ground with bandpass FSS, a transmission band appears at 1.65 THz without affecting the performance of absorbing band. As *E_F_* increases from 0 eV to 0.3 eV, the passband sustains high transmission over 0.7. Due to the controllable absorption along with the transmission window, the design can widen the application of TMA such as electromagnetic compatibility, stealth, and filtering relying on the anti-jamming capability and low loss for the normal signals. Although the transmission of the window decreases slightly with the increase of chemical potential, it can be avoided by further optimizing the structure to reduce the loss caused by the graphene layer. Our following work will focus on increasing the number of channels and explore the method to control the switch of the transmission band.
